# CK2 Down-Regulation Increases the Expression of Senescence-Associated Secretory Phenotype Factors through NF-κB Activation

**DOI:** 10.3390/ijms22010406

**Published:** 2021-01-02

**Authors:** Junbin Song, Young-Seuk Bae

**Affiliations:** School of Life Sciences, BK21 FOUR KNU Creative BioResearch Group, Kyungpook National University, Daegu 41566, Korea; junbin9292@naver.com

**Keywords:** SASP factors, NF-κB, SIRT1, AKT, protein kinase CK2, miRNA, anti-inflammatory agent

## Abstract

Senescent cells secrete pro-inflammatory factors, and a hallmark feature of senescence is senescence-associated secretory phenotype (SASP). The aim of this study is to investigate the protein kinase CK2 (CK2) effects on SASP factors expression in cellular senescence and organism aging. Here CK2 down-regulation induced the expression of SASP factors, including interleukin (IL)-1β, IL-6, and matrix metalloproteinase (MMP) 3, through the activation of nuclear factor-κB (NF-κB) signaling in MCF-7 and HCT116 cells. CK2 down-regulation-mediated SIRT1 inactivation promoted the degradation of inhibitors of NF-κB (IκB) by activating the AKT-IκB kinase (IKK) axis and increased the acetylation of lysine 310 on RelA/p65, an important site for the activity of NF-κB. *kin-10* (the ortholog of CK2β) knockdown increased *zmp-1*, *-2*, and *-3* (the orthologs of MMP) expression in nematodes, but AKT inhibitor triciribine and SIRT activator resveratrol significantly abrogated the increased expression of these genes. Finally, antisense inhibitors of miR-186, miR-216b, miR-337-3p, and miR-760 suppressed CK2α down-regulation, activation of the AKT-IKK-NF-κB axis, RelA/p65 acetylation, and expression of SASP genes in cells treated with lipopolysaccharide. Therefore, this study indicated that CK2 down-regulation induces the expression of SASP factors through NF-κB activation, which is mediated by both activation of the SIRT1-AKT-IKK axis and RelA/p65 acetylation, suggesting that the mixture of the four miRNA inhibitors can be used as anti-inflammatory agents.

## 1. Introduction

Cellular senescence is a program of arrested proliferation and altered gene expression caused by different types of stress. Although senescence has been regarded as an effective cancer suppression mechanism, it is also involved in some pathological conditions, such as aging, age-associated diseases, and tumorigenesis [1,2,3]. One possible mechanism of this pathological effect is the action of the senescence-associated secretory phenotype (SASP). SASP is associated with the expression of various cytokines and chemokines, including interleukin (IL)-1α/β, IL-6, IL-8, growth factors, such as epidermal growth factor and vascular endothelial growth factor, and matrix metalloproteinases (MMPs), such as MMP3 and MMP9 [4,5]. SASP affects senescent cells in an autocrine manner and the microenvironment surrounding senescent cells in a paracrine manner. SASP is; thus, becoming an attractive pharmacological target for manipulating senescence-mediated effects [6,7]. However, the molecular mechanism of SASP development remains elusive. The expression of SASP factors can be modulated by the transcription factor nuclear factor-κB (NF-κB), which is activated in senescent cells [8]. The most prevalent form of NF-κB exists as a heterodimer consisting of RelA/p65 and p50. NF-κB is normally retained in the cytosol by binding to inhibitors of NF-κB (IκB) [9]. Various upstream signaling cascades can activate the IκB kinase (IKK) complex, which subsequently phosphorylates IκBα. This leads to IκBα ubiquitination and proteasome-mediated degradation, allowing NF-κB to enter the nucleus and transcribe its target genes [10]. In addition, NAD^+^-dependent histone deacetylase, SIRT1, inhibits the transcriptional activity of NF-κB by deacetylating RelA/p65 at lysine 310 [11,12].

Protein kinase CK2 (CK2) is a ubiquitous serine/threonine kinase that catalyzes the phosphorylation of a large number of proteins. The holoenzyme of CK2 is a heterotetramer composed of two catalytic (α and/or α’) subunits and two regulatory β subunits. The β subunit stimulates the catalytic activity of the α or α’ subunit, thereby mediating tetramer formation and substrate recognition [13,14]. It has been shown that CK2 down-regulation induces several senescence markers, including senescence-associated β-galactosidase (SA-β-gal) activity, activation of the p53-p21^Cip1/WAF1^ axis, and senescence-associated heterochromatin foci (SAHF) formation [15,16,17]. CK2 down-regulation stabilizes p53 by accumulating reactive oxygen species (ROS) and inactivating SIRT1 deacetylase [18,19]. The activation of the phosphatidylinositol 3-kinase (PI3K)-AKT-mammalian target of rapamycin mTOR) pathway and the inactivation of transcription factors FoxO3a and Nrf2 are associated with ROS accumulation during CK2 down-regulation-mediated senescence [20,21,22]. miR-186, miR-216b, miR-337-3p, and miR-760 cooperatively promote cellular senescence by inhibiting CK2α [23,24]. In addition, CK2 activity decreased with advancing age in *Caenorhabditis elegans*, and *kin-10* (the ortholog of CK2β) knockdown led to a short lifespan phenotype and induced age-related biomarkers in worms [25]. Age-dependent CK2 down-regulation reduces longevity by associating with ROS generation via the AGE-1-AKT-1-DAF-16 pathway [23] and SIRT1-FoxO3a pathway [26] in *C. elegans*.

This study reported for the first time that CK2 down-regulation in human cancer cells enhances the expression of SASP factors (IL-1β, IL-6, and MMP3) by NF-κB activation through two pathways: an SIRT1-dependent/AKT-independent pathway and an SIRT1/AKT-dependent pathway. AKT and SIRT1 are also involved in the increased expression of *zmp-1, -2*, and *-3* (the orthologs of MMP) mediated by CK2 down-regulation in nematodes. Moreover, findings established the antisense inhibitors of miR-186, miR-216b, miR-337-3p, and miR-760 as anti-inflammatory agents.

## 2. Results

### 2.1. CK2 Down-Regulation Stimulates the Expression of SASP Factors by Enhancing the Nuclear Localization of NF-κB in Human Cancer Cells

**Figure 1:**
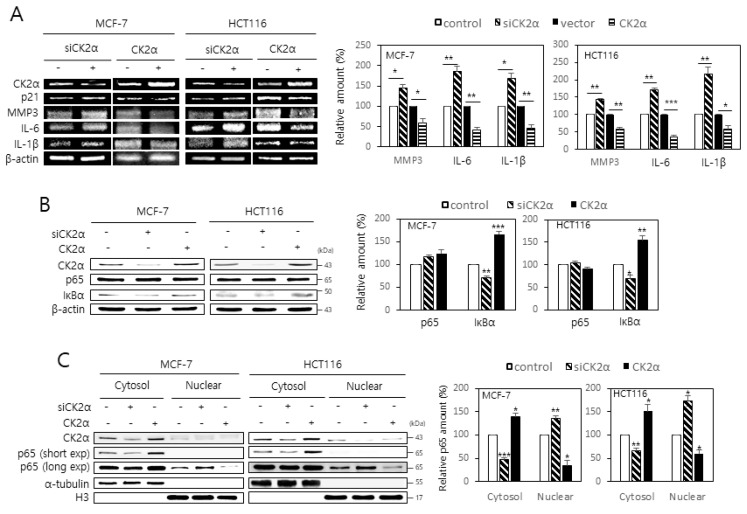
CK2 down-regulation stimulates the expression of senescence-associated secretory phenotype (SASP) factors by enhancing the nuclear localization of NF-kB in human cancer cells. MCF-7 and HCT116 cells were transfected with CK2α siRNA or pcDNA3.1-HA-CK2α for two days. (**A**) The level of each mRNA was measured by RT-PCR using gene-specific primers (**left**). Representative data from three independent experiments are shown. β-Actin was used as a control. Graphs represent the quantitation of each mRNA relative to β-actin (**right**). (**B**) The level of each protein was determined by immunoblot analysis using specific antibodies (**left**). Representative data from three independent experiments are shown. β-Actin was used as a control. Graphs represent the quantitation of each protein relative to β-actin (**right**). (**C**) Cytoplasm and nuclei were isolated from cells, and both extracts were visualized by immunoblotting. α-Tubulin (cytoplasmic marker) and histone H3 (nuclear marker) were quantified as loading controls (**left**). Representative data from three independent experiments are shown. Graphs represent the quantitation of RelA/p65 relative to the subcellular markers (**right**). exp, exposure. Data are mean ± standard error of the mean (SEM). * *p* < 0.05; ** *p* < 0.01; *** *p* < 0.001.

The role of CK2 on the expression of SASP factors in MCF-7 and HCT116 cells was investigated. Western blot analysis revealed that treatment with CK2α small interfering RNA (siRNA) increased the protein levels of SASP factors, including IL-6, IL-1β, and MMP3. Similarly, treatment with pcDNA-HA-CK2α decreased the expression of these factors (Supplementary Materials Figure S1). Consistent with previous reports [15,16], CK2α knockdown increased the levels of p53-p21^Cip1/WAF1^ protein. Whether CK2 regulates the mRNA levels of IL-6, IL-1β, and MMP3 was then determined. Reverse transcription-polymerase chain reaction (RT-PCR) analysis indicated that the mRNA levels of these factors were increased by CK2α down-regulation and decreased by CK2α overexpression compared to the control (Figure 1A). Thus, these data suggest that CK2 negatively regulates the expression of SASP factors at the transcriptional level in human cells. Because NF-κB is a major transcription factor in expressing SASP factors [6,8], whether CK2 regulates the protein levels of RelA/p65 and IκB was examined. The RelA/p65 protein levels remained unchanged after CK2α knockdown or overexpression. However, the protein levels of IκB were increased by CK2α down-regulation but decreased by CK2α overexpression compared to the control (Figure 1B). Because NF-κB is normally retained in the cytoplasm in a complex with IκB [6,8], cytoplasm and nuclei from cells transfected with CK2α siRNA or pcDNA-HA-CK2α were separated to examine the role of CK2 in the nuclear localization of RelA/p65. Increased accumulation of RelA/p65 was found in the nuclear extracts compared to the cytosolic extracts of CK2α-silenced cells. In contrast, increased accumulation of RelA/p65 was observed in the cytosolic extracts compared to the nuclear extracts of CK2α-overexpressing cells (Figure 1C). Collectively, these results suggest that CK2 down-regulation increases the nuclear import of NF-κB by down-regulating IκB, subsequently promoting SASP gene expression.

### 2.2. Activation of the AKT-IKK-IκB Pathway Is Associated with the CK2 Down-Regulation-Mediated Nuclear Import of NF-κB

**Figure 2:**
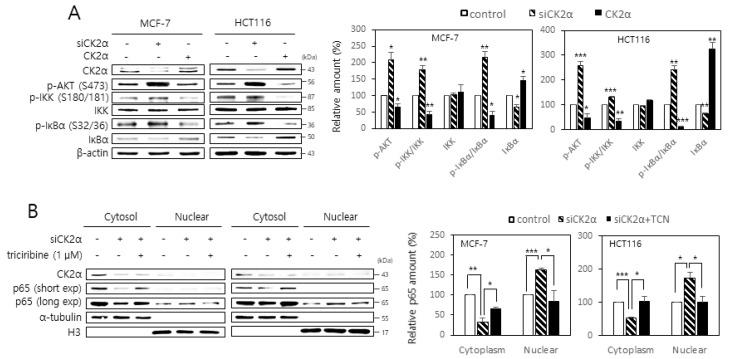
Activation of the AKT- IκB kinase (IKK)-inhibitors of NF-κB (IκB) pathway is associated with the CK2 down-regulation-mediated nuclear import of NF-κB. (**A**) MCF-7 and HCT116 cells were transfected with CK2α siRNA or pcDNA3.1-HA-CK2α for two days. The level of each protein was determined by immunoblot analysis using specific antibodies (**left**). Representative data from three independent experiments are shown. β-Actin was used as a control. Graphs represent the quantitation of p-AKT, IKK, and IκBα relative to β-actin and that of p-IKK and p-IκBα relative to the unphosphorylated proteins (**right**). (**B**) Cells were transfected with CK2α siRNA or pcDNA3.1-HA-CK2α for two days in the absence or presence of 1 μM triciribine (TCN). Cytoplasm and nuclei were isolated from cells, and both extracts were visualized by immunoblotting. α-Tubulin (cytoplasmic marker) and histone H3 (nuclear marker) were quantified as loading controls (**left**). Representative data from three independent experiments are shown. Graphs represent the quantitation of RelA/p65 relative to the subcellular markers (**right**). exp, exposure. Data are mean ± SEM. * *p* < 0.05; ** *p* < 0.01; *** *p* < 0.001.

Because IKK is known to phosphorylate and reduce the stability of IκB [10], whether CK2 regulates the activity of IKK was examined by monitoring the phosphorylation of serine residues 32 and 36 on IκBα. CK2 down-regulation increased the phosphorylation of these residues on IκBα, whereas CK2α overexpression decreased their phosphorylation (Figure 2A). Because AKT stimulates IKK activity by phosphorylating serine 180 on IKK [10], whether CK2 regulates AKT-mediated IKK phosphorylation was investigated by monitoring the phosphorylation of serine 180 on IKK. Whereas CK2 down-regulation increased IKK phosphorylation, CK2α overexpression decreased its phosphorylation (Figure 2A). Furthermore, treatment with the AKT inhibitor, triciribine (1 μM), attenuated the AKT, IKK, and IκBα phosphorylation mediated by CK2 down-regulation (Figure S2). Whether AKT was involved in the CK2 down-regulation-mediated nuclear import of NF-κB and SASP gene expression was then determined. Treatment with triciribine (1 μM) both attenuated the nuclear import of RelA/p65 and increased the mRNA levels of SASP factors induced by CK2 down-regulation (Figure 2B; Figure S3). These results collectively demonstrate that the nuclear import of NF-κB and the subsequent expression of SASP factors are mediated by the activation of the AKT-IKK-IκB signaling axis in CK2-down-regulated cells.

### 2.3. SIRT1 Attenuates both RelA/p65 Acetylation and Activation of the AKT-IKK-IκB Axis Mediated by CK2 Down-Regulation

**Figure 3:**
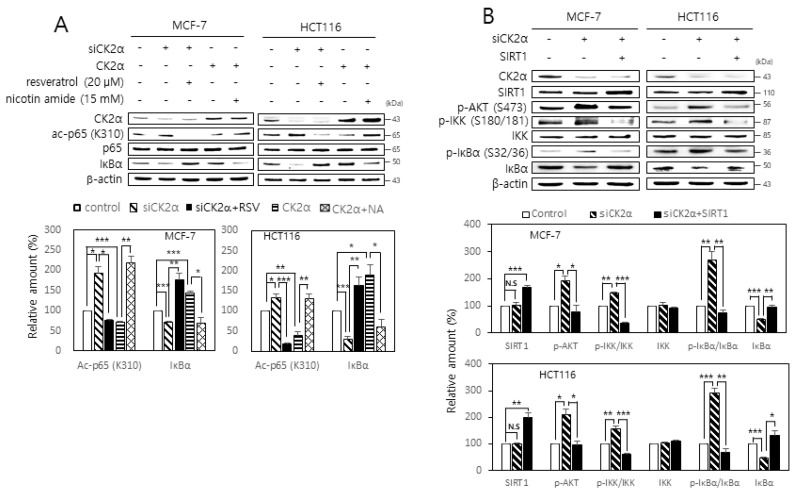
SIRT1 attenuates both RelA/p65 acetylation and activation of the AKT-IKK-IκB axis mediated by CK2 down-regulation. (**A**) MCF-7 and HCT116 cells were transfected with CK2α siRNA or pcDNA3.1-HA-CK2α for two days in the absence or presence of 20 μM resveratrol or 15 mM nicotine amide. The level of each protein was determined by immunoblot analysis using specific antibodies (**top**). Representative data from three independent experiments are shown. β-Actin was used as a control. Graphs represent the quantitation of acetylated p65 (ac-p65) relative to unacetylated p-65 (**bottom**). RSV, resveratrol; NA, nicotine amide. (**B**) Cells were transfected with CK2α siRNA and/or pECE-Flag-SIRT1 for two days. The level of each protein was determined by immunoblot analysis using specific antibodies (**top**). Representative data from three independent experiments are shown. β-Actin was used as a control. Graphs represent the quantitation of p-AKT, IKK, and IκBα relative to β-actin and that of p-IKK and p-IκBα relative to unphosphorylated proteins (**bottom**). Data are mean ± SEM. * *p* < 0.05; ** *p* < 0.01; *** *p* < 0.001.

SIRT1 mediates the deacetylation of lysine residue 310 on RelA/p65 [11,12]. Therefore, whether CK2 regulates SIRT1-mediated RelA/p65 deacetylation was examined. Whereas CK2 down-regulation increased RelA/p65 acetylation, CK2α overexpression decreased its acetylation. Furthermore, treatment with the SIRT1 activator, resveratrol (20 μM), attenuated the increase in RelA/p65 acetylation induced by CK2 down-regulation, whereas treatment with the SIRT1 inhibitor, nicotine amide (15 mM), restored the decrease in RelA/p65 acetylation induced by CK2 overexpression (Figure 3A). Next, the role of SIRT1 in IκB activation mediated by CK2 down-regulation was examined. Treatment with resveratrol (20 μM) rescued the CK2 down-regulation-mediated decrease in IκB. Treatment with nicotine amide (15 mM) attenuated the CK2 up-regulation-mediated increase in the IκB amount (Figure 3A). Furthermore, SIRT1 overexpression or treatment with resveratrol (20 μM) attenuated both AKT, IKK, and IκBα phosphorylation and IκBα suppression mediated by CK2 down-regulation (Figure 3B; Figure S2). Treatment with resveratrol (20 μM) attenuated the increase in SASP gene expression levels induced by CK2 down-regulation (Figure S3). Taken together, these data suggest that CK2 down-regulation stimulates SASP gene expression through both increased RelA/p65 acetylation and activation of the AKT-IKK-IκB axis mediated by SIRT1 inhibition.

### 2.4. Triciribine and Resvzeratrol Attenuate the Expression of MMP Orthologs Induced by CK2 Down-Regulation in Nematodes

**Figure 4:**
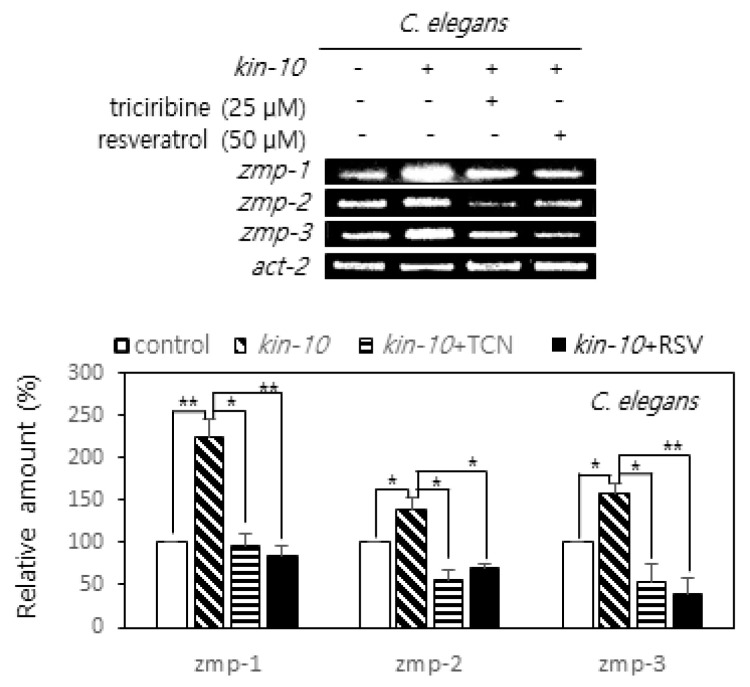
Effect of triciribine and resveratrol on the expression of matrix metalloproteinases (MMPs) orthologs induced by CK2 down-regulation in nematodes. Age-synchronized worms at the L4 stage were fed on the control or kin-10 RNAi plates containing 25 μM triciribine or 50 μM resveratrol for three days. Lysates from nematodes were utilized in RT-PCR using specific primers. PCR products were resolved on a 1.5% agarose gel. Representative data from three independent experiments are shown (**top**). Representative data from three independent experiments are shown. Act-2 mRNA served as the loading control. Graphs represent the quantitation of the mRNA levels of each gene relative to that of act-2 (**bottom**). TCN, triciribine; RSV, resveratrol. Data are mean ± SEM. * *p* < 0.05; ** *p* < 0.01.

*C. elegans* has been widely used as a model for exploring the mechanisms underlying aging. CK2 down-regulation in nematodes resulted in reduced longevity and onset of age-related biomarkers associated with both AGE-1/PI3K-AKT-1/AKT-DAF-16/FoxO and SIR-2.1/SIRT1-DAF-16/FoxO3a axes [25,26]. To investigate the effect of CK2 down-regulation on the expression of SASP factors in worms, the expression of *zmp-1* (the ortholog of MMP17 and MMP21), *zmp-2* (the ortholog of MMP21), and *zmp-3* (the ortholog of MMP1, MMP8, and MMP25) in nematodes treated with *kin-10* (the ortholog of CK2β) RNA interference (RNAi) was compared to that in nematodes treated with empty vector control (L4440) RNAi. *kin-10* knockdown increased *zmp-1*, *-2*, and *-3* expression in nematodes. To analyze the role of AKT and SIRT1 in *zmp-1*, *zmp-2*, and *zmp-3* expression in *C. elegans* after *kin-10* knockdown, nematodes were further treated with triciribine (25 μM) or resveratrol (50 μM). Treatment with triciribine or resveratrol significantly abrogated the increased expression of *zmp-1*, *-2*, and *-3* induced by *kin-10* knockdown (Figure 4). Therefore, these results suggest that AKT and SIRT1 are involved in the increased expression of *zmp-1*, *zmp-2*, and *zmp-3* mediated by CK2 down-regulation in nematodes.

### 2.5. Antisense Inhibitors of miR-186, miR-216b, miR-337-3p, and miR-760 Attenuate SASP Gene Expression Induced by Lipopolysaccharide (LPS)

**Figure 5:**
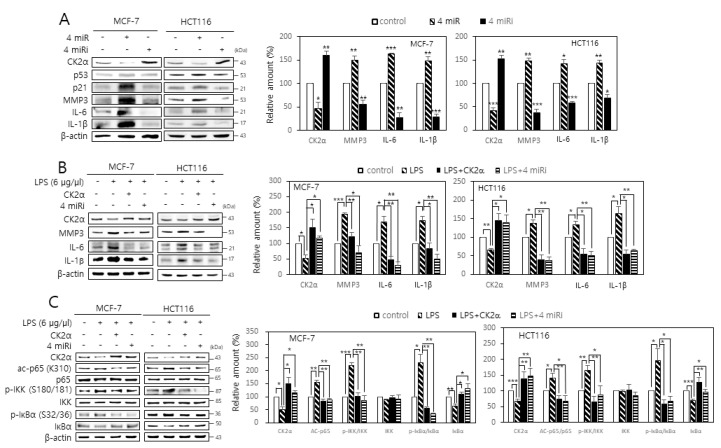
Antisense inhibitors of miR-186, miR-216b, miR-337-3p, and miR-760 attenuate the induction of senescence-associated secretory phenotype (SASP) factors by lipopolysaccharide (LPS). (**A**) MCF-7 and HCT116 cells were transfected with the 4 miRs (miR-186, miR-216b, miR-337-3p, and miR-760) or the 4 miRi. (**B**,**C**) Cells were treated with LPS (6 μg/μL) in the presence or absence of the 4 miRi or pcDNA3.1-HA-CK2α for 2 days. The level of each protein was determined by immunoblot analysis using specific antibodies (**left**). Representative data from three independent experiments are shown. β-Actin was used as a control. Graphs represent the quantitation of p-AKT, IKK, and IκBα relative to β-actin and that of p-IKK and p-IκBα relative to the unphosphorylated proteins (**right**). Data are mean ± SEM. * *p* < 0.05; ** *p* < 0.01; *** *p* < 0.001.

It has been shown that miR-186, miR-216b, miR-337-3p, and miR-760 cooperatively promote cellular senescence by targeting CK2α, whereas antisense inhibitors of these four miRNAs (4 miRs) suppressed CK2 inhibition-mediated senescence by up-regulating CK2α [23,24]. Here, the effects of these 4 miRs and antisense inhibitors on SASP gene expression in human cancer cells were investigated. Treatment with the 4 miRs increased SASP expression, whereas treatment with the four miR inhibitors (4 miRi) resulted in decreased SASP expression (Figure 5A). Because LPS induces cellular senescence in multiple cell types [27,28], the effect of the 4 miRi on SASP gene expression in cells treated with LPS was examined. Although treatment with LPS (6 μg/μL) increased SASP expression and decreased CK2α expression in human cancer cells, additional treatment with the 4 miRi or CK2α overexpression abrogated the LPS-mediated induction of SASP factors (Figure 5B). Furthermore, treatment with the 4 miRi or CK2α overexpression suppressed RelA/p65 acetylation and IKK and IκBα phosphorylation, which were mediated by LPS (Figure 5C). These results collectively indicate that antisense inhibitors of miR-186, miR-216b, miR-337-3p, and miR-760 decrease SASP factors by inhibiting NF-κB signaling, suggesting that the 4 miRi together may act as an effective anti-inflammatory agent. Finally, the effect of individual miR inhibitors on the LPS-mediated induction of SASP gene expression was investigated. Each of the 4 miRi suppressed the LPS-mediated induction of SASP factors, but this effect was more strongly suppressed by the mixture of the four miRNA inhibitors compared to individual treatment (Figure S4).

## 3. Discussion

**Figure 6:**
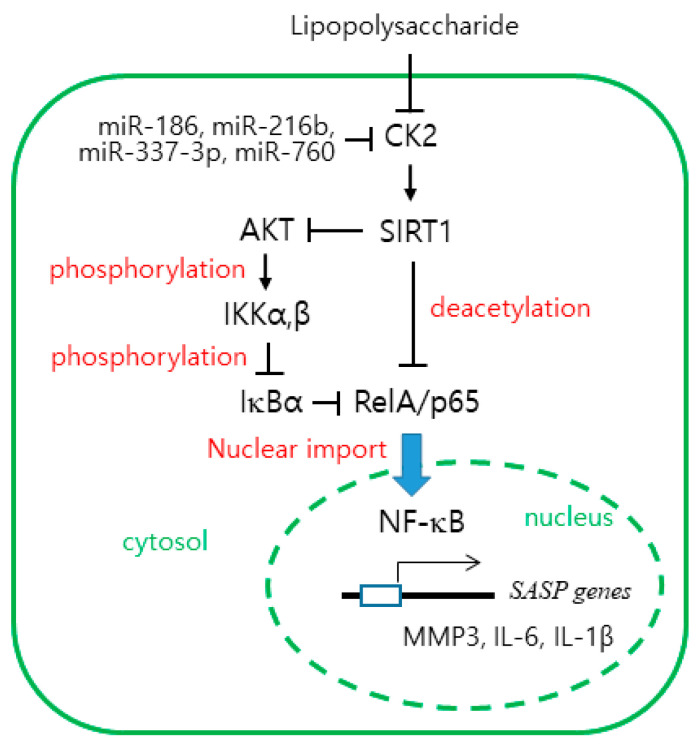
The working model for the CK2 down-regulation-mediated expression of SASP factors. CK2 down-regulation induces SIRT1 inhibition and AKT up-regulation, which elevate the nuclear import of RelA/p65 by increasing RelA/p65 acetylation and IκB destabilization, respectively. Subsequently, active NF-κB stimulates the transcription of SASP genes, including MMP2, IL-6, and IL-1β. LPS down-regulates CK2. The antisense inhibitors of miR-186, miR-216b, miR-337-3p, and miR-760 exhibit anti-inflammatory effects.

Senescent cells secrete pro-inflammatory proteins known as SASP factors [4,5,6]. NF-κB is the major regulator that induces the appearance of SASP [8,9]. CK2 down-regulation was previously reported to induce cellular senescence and organism aging [15,16,17,18,19,20,21,22,23,24,25,26], but its relationship to SASP factors is unknown. In this study, we examined the role of CK2 in the expression of SASP. The present study shows that CK2 down-regulation induces the nuclear import of NF-κB by reducing the IκB levels, which promotes the subsequent expression of SASP factors, such as IL-6, IL-1β, and MMP3 in human cancer cells. In contrast, CK2 up-regulation decreases the nuclear import of NF-κB by increasing the IκB levels and reduces the expression of SASP factors, suggesting that CK2 negatively regulates NF-κB signaling (Figure 1; Figure S1). This study demonstrated that CK2 regulates NF-κB signaling through two pathways. First, CK2 regulates NF-κB activity through the AKT-IKK-IκB pathway. CK2 down-regulation induced an increase in nuclear NF-κB and the phosphorylation of AKT, IKK, and IκB, which was reversed by treatment with the AKT inhibitor, triciribine (Figure 2; Figure S2). Similarly, treatment with triciribine rescued the CK2 down-regulation-mediated induction of SASP factors in human cells (Figure S3) and *kin-10* knockdown-mediated induction of MMP orthologs in nematodes (Figure 4). Because IKK promotes the ubiquitin-dependent degradation of IκBα by phosphorylating IκBα [10], this study suggests that CK2 down-regulation induces IκBα degradation and subsequent NF-κB nuclear localization through the activation of the AKT-IKK pathway (Figure 6).

Second, CK2 regulates NF-κB activity through SIRT1. Whereas CK2 down-regulation increased RelA/p65 acetylation, treatment with the SIRT1 activator, resveratrol, abrogated the increase in RelA/p65 acetylation induced by CK2 down-regulation. In contrast, whereas CK2α overexpression decreased its acetylation, treatment with the SIRT1 inhibitor, nicotine amide, suppressed the decrease in RelA/p65 acetylation induced by CK2 up-regulation (Figure 3A). Furthermore, SIRT1 overexpression or treatment with resveratrol attenuated the AKT-IKK axis-dependent IκBα degradation mediated by CK2 down-regulation, suggesting that SIRT1 acts as a negative regulator of AKT-IKK-NF-κB signaling (Figure 3B; Figure S2). Likewise, resveratrol treatment rescued the CK2 down-regulation-induced induction of SASP factors in human cells (Figure S3), and *kin-10* knockdown-mediated an increase in the MMP orthologs in nematodes (Figure 4). Taken together, these results suggest that SIRT1 is involved in CK2 down-regulation-mediated SASP induction through RelA/p65 deacetylation and AKT activation (Figure 6). This conclusion is consistent with the results from our previous reports showing that CK2 down-regulation up-regulates several senescence markers (increased SA-β-gal activity, ROS production, activation of the p53-p21^Cip1/WAF1^ axis, and SAHF formation) by activating the PI3K-AKT-mTOR pathway and inactivating SIRT1 [15,16,17,18,19,20,21,22]. Chai et al. [29] have recently shown that resveratrol inhibits the PI3K-AKT pathway by SIRT1 activation. Collectively, we conclude that CK2 functions as a negative regulator of NF-κB in senescent cells. This is not consistent with previous reports that CK2 induces NF-κB activation at multiple levels through IκB [30], inducible IKK and IKKε [31], IKK2 [32], or RelA/p65 itself [33,34]. Although we currently cannot explain this discrepancy, we hypothesize that CK2 may regulates NF-κB activity by different mechanisms depending on the state of cell growth (e.g., proliferation or senescence).

These results indicate that antisense inhibitors of miR-186, miR-216b, miR-337-3p, and miR-760 exhibit anti-inflammatory effects. These four miRNA inhibitors decreased the expression of SASP factors by up-regulating CK2. Furthermore, these four miRNA inhibitors successfully abrogated the LPS-mediated induction of SASP factors, RelA/p65 acetylation, and activation of the AKT-IKK axis. Interestingly, treatment with LPS decreased the level of CK2α protein (Figure 5 and Figure 6). For many years, CK2 was thought to be a constitutive, nonregulated protein kinase [13,14]. Our recent reports have shown that CK2 is downregulated in replicative or oxidative stress-induced senescence in human cells and in nematode aging [15,16,17,18,19,20,21,22,23,24,25,26]. The present study also indicates that LPS downregulates CK2 expression. However, how LPS down-regulates CK2α currently cannot be explained. Among the 4 miRs, it has been reported that miR-186 and miR-216b are associated with inflammation. miR-186 promotes the secretion of pro-inflammatory cytokines by targeting cystathionine γ-lyase in THP-1 macrophages [35]. miR-216b down-regulation inhibits IL-1β-induced chondrocyte injury by up-regulating Smad3 [36]. This study reports, for the first time, the anti-inflammatory effects of antisense inhibitors of miR-216b and miR-337-3p.

It has been shown that the high mobility group (HMG) B2 protein orchestrates SASP through the prevention of heterochromatin spreading to exclude SASP gene loci from senescence-associated heterochromatin [37]. In addition, the CK2-mediated phosphorylation of the acidic tail of HMGB2 modulates the intranuclear distribution of HMGB2 [38]. A previous study demonstrated that CK2 down-regulation promotes SAHF formation by enhancing H3K9 trimethylation [17]. Because CK2 down-regulation induces the expression of SASP factor genes in this study, it is proposed that CK2 down-regulation may allow for the exclusion of SASP gene loci from SAHF by reducing phosphorylation on the acidic tail of HMGB2 in senescent cells.

In conclusion, these findings establish CK2 as a key switch to regulate SASP factors. The results suggest that SIRT1 connects CK2 down-regulation to SASP factors through NF-κB activation, which is mediated by both RelA/p65 deacetylation and activation of the AKT-IKK-IκB axis. Because senescence is thought to promote aging-associated diseases through the resulting inflammatory response, the four inhibitors of miR-186, miR-216b, miR-337-3p, and miR-760 may provide a strategy for anti-inflammatory intervention.

## 4. Materials and Methods

### 4.1. Materials

Antibodies against SIRT1, CK2α, p53-p21^Cip1/WAF1^, RelA/p65, IκB, α-tubulin, and β-actin were obtained from Santa Cruz Biotechnology (Santa Cruz, CA, USA). Antibodies against acetylated RelA/p65 (ac-p65, K310), IKKα/β, phosphorylated IKKα/β (p-IKKα/β, S180/S181), IL-1β, IL-6, MMP3, and histone H3 were obtained from Abcam (Cambridge, UK). Antibodies against phosphorylated IκBα (p-IκBα, S32/S36) and phosphorylated AKT (p-AKT, S473) were obtained from Cell Signaling (Danvers, MA, USA). Resveratrol, triciribine, nicotine amide, and LPS were obtained from the Sigma Chemical Co. (St. Louis, MO, USA).

### 4.2. Cell Culture, RNAi, and DNA Transfection

Human breast cancer MCF-7 cells and human colon cancer HCT116 cells (American Type Culture Collection, Manassas, VA, USA) were cultured in Dulbecco’s modified Eagle’s medium containing 10% (*v*/*v*) fetal bovine serum and 1% (*v*/*v*) penicillin-streptomycin in a humidified atmosphere of 95% (*v*/*v*) air and 5% (*v*/*v*) CO_2_ at 37 °C. Cells were transfected with pcDNA3.1-HA-CK2α or pECE-Flag-SIRT1 using Polyfect (Qiagen, Hilden, Germany) according to the manufacturer’s instructions. Mimics for miR-186, miR-216b, miR-337-3p, and miR-760 and control miRNA were purchased from Genolution, Inc. (Seoul, Korea). Antisense inhibitors for the 4 miRs were obtained from Panagene, Inc. (Seoul, Korea). The siRNA for CK2α was 5′-GAUGACUACCAGCUGGUUCdTdT-3′. The siRNA for the negative control was 5′-GCUCAGAUCAAUACGGAGAdTdT-3′. RNAs were transfected into cells using Lipofectamine (Invitrogen, Carlsbad, CA, USA) for 48 h.

### 4.3. Immunoblotting

Proteins were separated on 10% sodium dodecyl sulfate-polyacrylamide gels and then transferred by electrophoresis onto nitrocellulose membranes. The membranes were blocked with 5% (*w/v*) non-fat, dry, skim milk dissolved in TBST [20 mM Tris-HCl (pH 7.4), 150 mM NaCl, 0.05% Tween 20] for 2 h and then incubated with specific antibodies diluted in TBST with 1% (*w/v*) non-fat, dry, skim milk for 1 h. The membranes were washed three times in TBST and then analyzed with an ECL system (Amersham, UK).

### 4.4. RT-PCR

Total RNA was extracted from human cancer cells or nematodes. RNA was reverse transcribed using gene-specific primers and reverse transcriptase (Takara Bio, Inc., Kyoto, Japan), and the resulting cDNAs were amplified by PCR. The primers used for the assays are listed in Table S1. PCR products were resolved on a 1.5% agarose gel. The quantitation of the RT-PCR bands was performed using densitometry. β-Actin RNA levels were used to standardize the amounts of RNA in each sample.

### 4.5. Isolation of Nuclear and Cytoplasmic Extracts

The cytoplasmic and nuclear extracts were prepared using an NE-PER Nuclear Cytoplasmic Extraction Reagent kit (Pierce, Rockford, IL, USA) according to the manufacturer’s instructions.

### 4.6. Culture of Nematodes and RNAi Experiment

The *C. elegans* N2 strain was acquired from the Caenorhabditis Genetics Center. Nematodes were grown at 21 °C on nematode growth medium agar plates with *Escherichia coli* strain OP50 as a food source. For some experiments, nematodes were treated with triciribine or resveratrol. RNAi experiments were performed with *E. coli* HT115 cells expressing double-stranded *kin-10* RNA, as described previously [25,26].

### 4.7. Statistical Analysis

Data were analyzed by one-way analysis of variance with the SPSS package program (IBM, Armonk, NY, USA). The results were considered significant if *p* < 0.05. Duncan’s multiple-range test was performed if significant differences between the groups were identified using α = 0.05.

## Data Availability

Not applicable.

## Supplementary Materials

Supplementary Materials can be found at https://www.mdpi.com/1422-0067/22/1/406/s1.

## Abbreviations

CK2	protein kinase CK2
HMG	high mobility group
IκB	inhibitors of NF-κB
IKK	IκB kinase
IL	interleukin
LPS	lipopolysaccharide
mTOR	mammalian target of rapamycin
MMP	matrix metalloproteinase
NF-κB	nuclear factor-κB
RT-PCR	reverse transcription-polymerase chain reaction
PI3K	phosphatidylinositol 3-kinase
ROS	reactive oxygen species
SA-β-gal	senescence-associated β-galactosidase
SAHF	senescence-associated heterochromatin foci
SASP	senescence-associated secretory phenotype
siRNA	small interfering RNA
