# Supplementary material for: CK2 Down-Regulation Increases the Expression of Senescence-Associated Secretory Phenotype Factors through NF-κB Activation
Source: Int J Mol Sci. 2021 Jan 2;22(1):406. doi: 10.3390/ijms22010406 (PMC7795172; doi:10.3390/ijms22010406)
Supplement: Supplementary file 1 [file ijms-22-00406-s001.zip › Supplementary files/Supplemental Figure legends.docx]

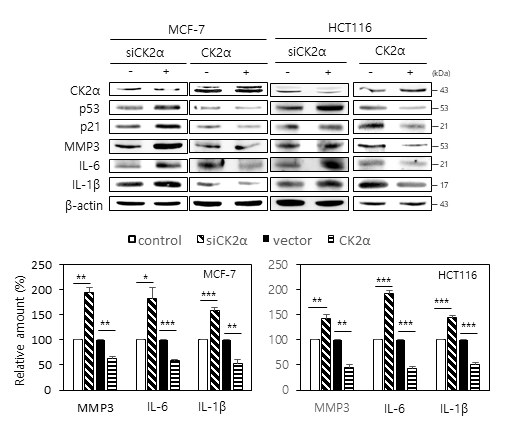


**Figure S1.** CK2 down-regulation stimulates the protein levels of SASP factors in human cancer cells. MCF-7 and HCT116 cells were transfected with CK2α siRNA or pcDNA3.1-HA-CK2α for 2 days. The level of each protein was determined by immunoblot analysis using specific antibodies (top). Representative data from three independent experiments are shown. β-Actin was used as a control. Graphs represent the quantitation of each protein relative to β-actin (bottom). Data are mean ± SEM. **P* < 0.05; ***P* < 0.01; ****P* < 0 .001.


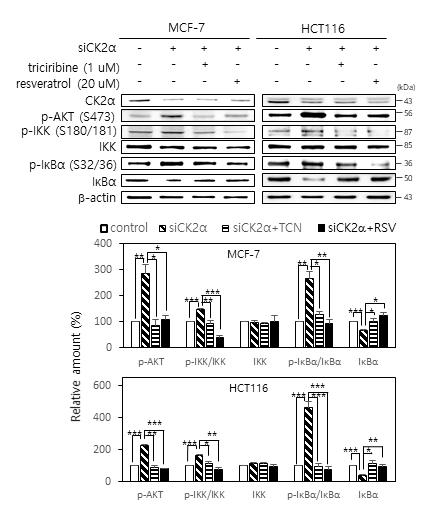


**Figure S2.** Effect of triciribine and resveratrol on phosphorylation of AKT, IKK and IκBα induced by CK2 down-regulation in human cancer cells. MCF-7 and HCT116 cells were transfected with CK2α siRNA for 2 days in the absence or presence of 1 μM triciribine or 20 μM resveratrol. The level of each protein was determined by immunoblot analysis using specific antibodies (top). Representative data from three independent experiments are shown. β-Actin was used as a control Graphs represent the quantitation of p-AKT, IKK, and IκBα relative to β-actin and that of p-IKK and p-IκBα relative to the unphosphorylated proteins (bottom). TCN, triciribine; RSV, resveratrol. Data are mean ± SEM. **P* < 0.05; ***P* < 0.01; ****P* < 0 .001.


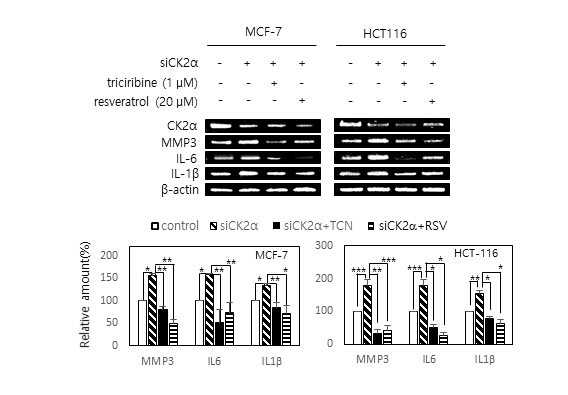


**Figure S3.** Effect of triciribine and resveratrol on expression of SASP factors induced by CK2 down-regulation in human cancer cells. MCF-7 and HCT116 cells were transfected with CK2α siRNA for 2 days in the absence or presence of 1 μM triciribine or 20 μM resveratrol. Total RNA was extracted from cells and reverse-transcribed using CK2α-specific primers and reverse transcriptase. PCR products were resolved on a 1.5% (w/v) agarose gel (top). Representative data from three independent experiments are shown. β-Actin was used as a control. Graphs represent the quantitation of each protein relative to β-actin (bottom). TCN, triciribine; RSV, resveratrol. Data are mean ± SEM. **P* < 0.05; ***P* < 0.01; ****P* < 0 .001.


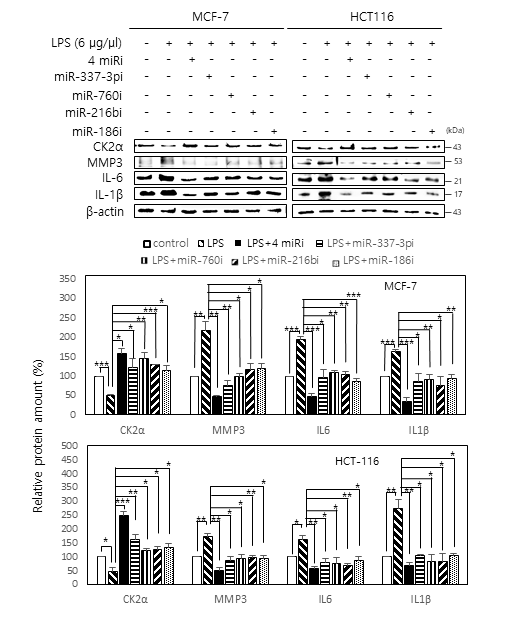


**Figure S4.** Effect of individual four miRNA inhibitors on LPS-mediated induction of SASP gene expression. MCF-7 and HCT116 cells were transfected with the four miRNA inhibitors mixture (4 miRi) or individual four miR inhibitors (miR-186i, miR-216bi, miR-337-3pi, and miR-760i) in the presence or absence of LPS (6 μg/μl) for 2 days. The level of each protein was determined by immunoblot analysis using specific antibodies (top). Representative data from three independent experiments are shown. β-Actin was used as a control. Graphs represent the quantitation of each protein relative to β-actin (bottom). Data are mean ± SEM. *P < 0.05; **P < 0.01; ***P < 0 .001.
