# Supplementary material for: CK2 Down-Regulation Increases the Expression of Senescence-Associated Secretory Phenotype Factors through NF-κB Activation
Source: Int J Mol Sci. 2021 Jan 2;22(1):406. doi: 10.3390/ijms22010406 (PMC7795172; doi:10.3390/ijms22010406)
Supplement: Supplementary file 1 [file ijms-22-00406-s001.zip › Supplementary files/Table S1.docx]

Supplemental Table S1. Primers used for RT-PCR analyses

| Gene |  | Primer sequence |
| --- | --- | --- |
| CK2α (h)  IL-1β (h)  IL-6 (h)  MMP3 (h)  p21 (h)  β-actin (h)  *zmp-1* (n)  *zmp-2* (n)  *zmp-3* (n)  *act-2* (n) | Forward  Reverse  Forward  Reverse  Forward  Reverse  Forward  Reverse  Forward  Reverse  Forward  Reverse  Forward  Reverse  Forward  Reverse  Forward  Reverse  Forward  Reverse | 5′-AAGACCCTGTGTCACGAACCC-3′  5′-GGCTCCTCCCGAAAGATCATAC-3′  5′-AGTGGCAATGAGGATGACTTGT-3′  5′-AGATGAAGGGAAAGAAGGTGCT-3′  5′-CACACAGACAGCCACTCACC-3′  5′-AGGTTGTTTTCTGCCAGTGC-3′  5′-CTCACAGACCTGACTCGGTT-3′  5′-CACGCCTGAAGGAAGAGATG-3′  5′-ATGGAACTTCGACTTTGTCA-3′  5′-GCTTCCTCTTGGAGAAGATC-3′  5′-TCCCTGGAGAAGAGCTACGA-3′  5′-AGCACTGTGTTGGCGTACAG-3′  5′-TCAAGCAGCTTCTCAGTGGA-3′  5′-TTTGTCGGGAAGAAAGCGTG-3′  5′-AGCTCGAGGACCAACAAGAA-3′  5′-AAATGCAAGCCGAGTGTGAG-3′  5′-TGTCAATGCTGCTCTCTGGA-3′  5′-CTGATGCCCGTTAATCCAGC-3′  5′-CCCAATTGAGCATGGTATCG-3′  5′-AGCGTTCGTTTCCAACAGTG-3′ |

h, human; n, nematode
