# Supplementary figures and images for: CK2 Down-Regulation Increases the Expression of Senescence-Associated Secretory Phenotype Factors through NF-κB Activation
Source: Int J Mol Sci. 2021 Jan 2;22(1):406. doi: 10.3390/ijms22010406 (PMC7795172; doi:10.3390/ijms22010406)

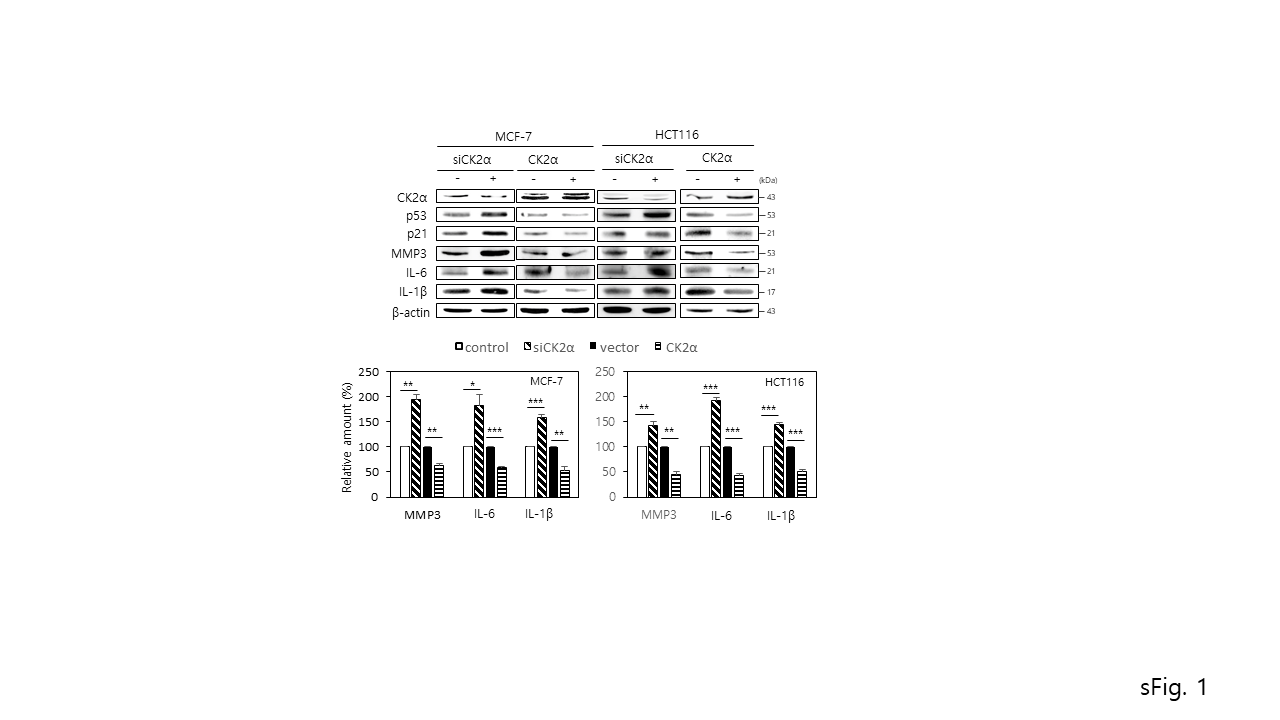

Supplement: Supplementary file 1 [file ijms-22-00406-s001.zip › Supplementary files/sFig. 1.tif]

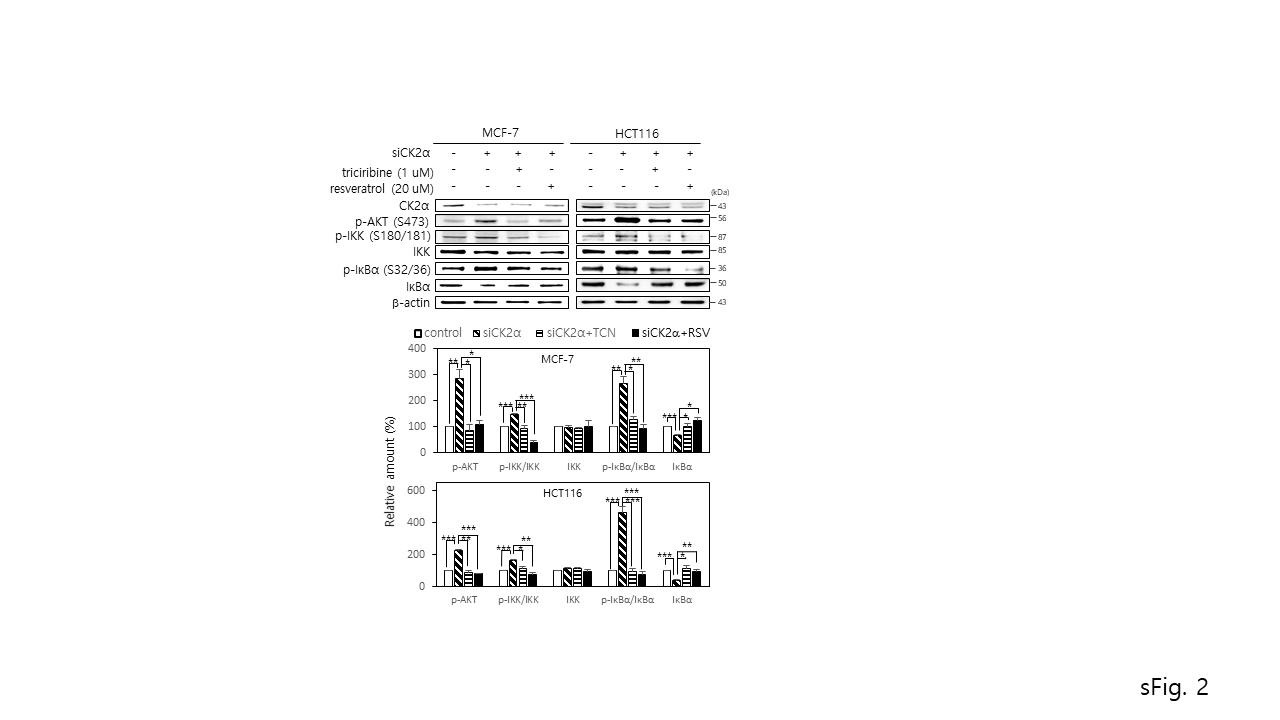

Supplement: Supplementary file 1 [file ijms-22-00406-s001.zip › Supplementary files/sFig. 2.tif]

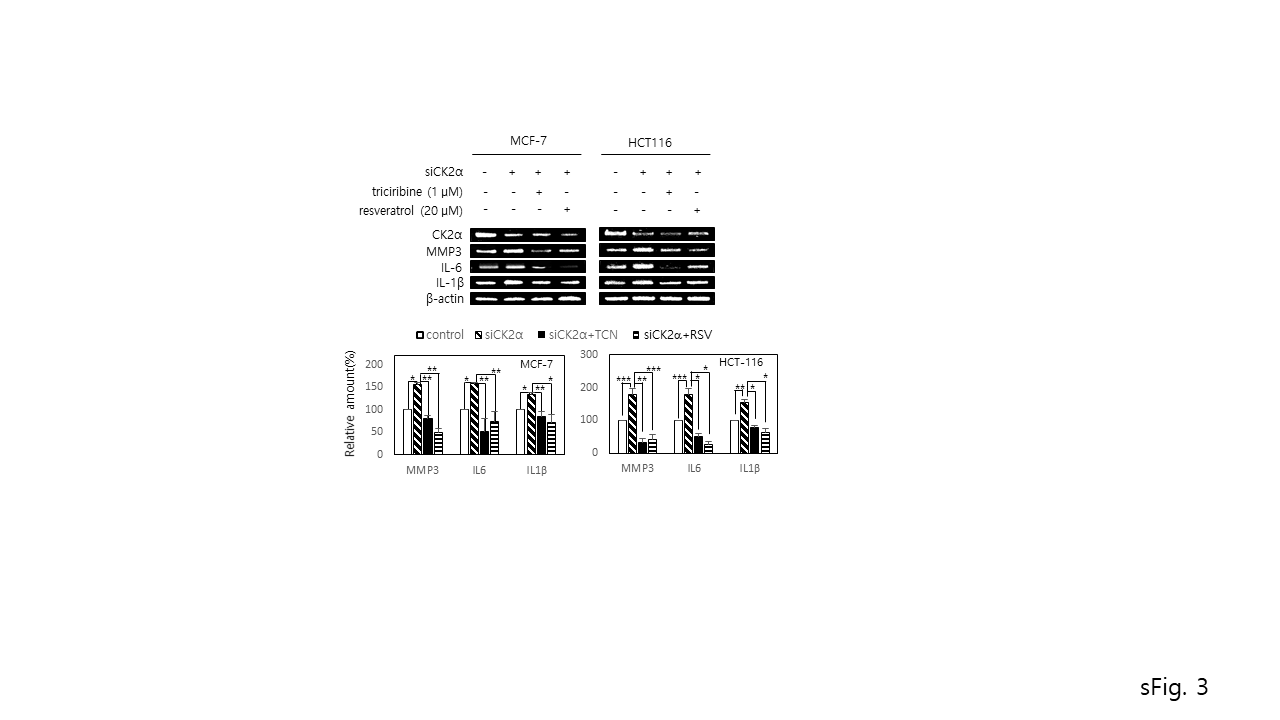

Supplement: Supplementary file 1 [file ijms-22-00406-s001.zip › Supplementary files/sFig. 3.tif]

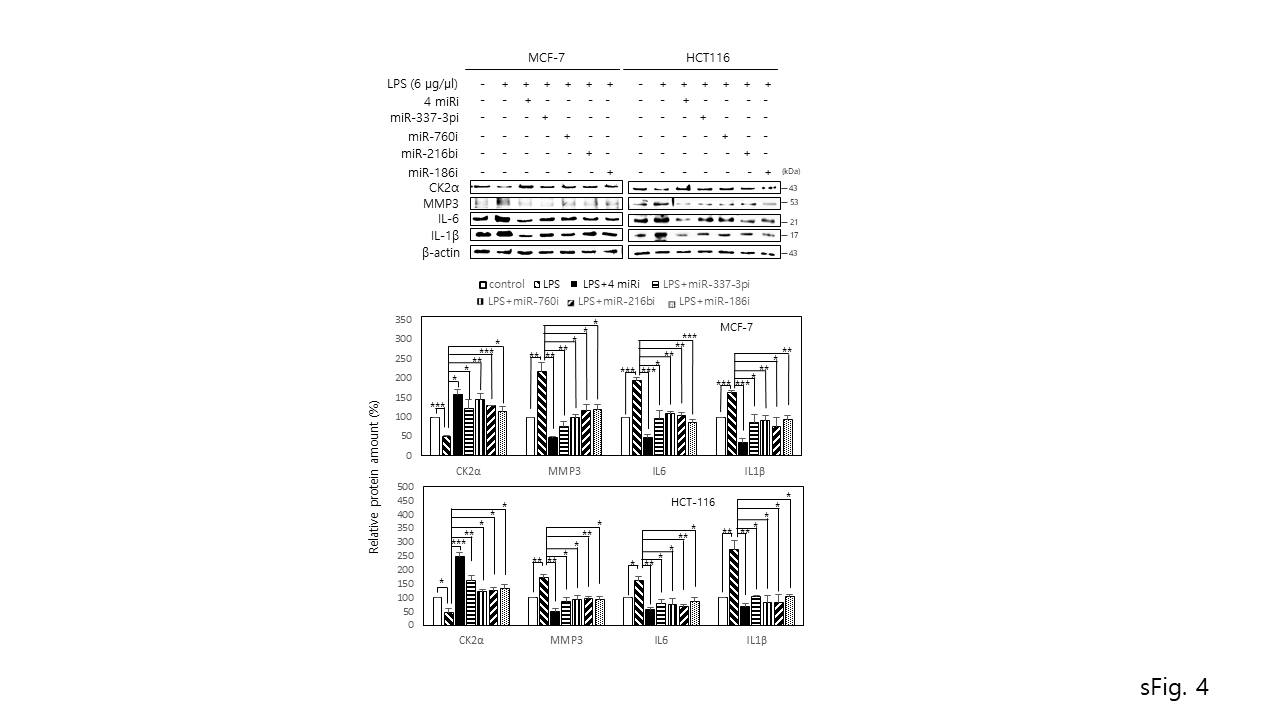

Supplement: Supplementary file 1 [file ijms-22-00406-s001.zip › Supplementary files/sFig. 4.tif]
